# Race modifies survival benefit of guideline‐based treatment: Implications for reducing disparities in muscle invasive bladder cancer

**DOI:** 10.1002/cam4.3429

**Published:** 2020-09-01

**Authors:** Samuel L. Washington, Steven E. Gregorich, Maxwell V. Meng, Anne M. Suskind, Sima P. Porten

**Affiliations:** ^1^ Department of Urology University of California San Francisco CA USA; ^2^ School of Medicine University of California San Francisco CA USA

**Keywords:** African continental ancestry group, cohort studies, social determinants of health, United States, urinary bladder neoplasms

## Abstract

**Background:**

Black individuals with muscle‐invasive bladder cancer (MIBC) experienced 21% lower odds of guideline‐based treatment (GBT) and differences in treatment explain 35% of observed Black‐White differences in survival. Yet little is known of how interactions between race/ethnicity and receipt of GBT drive within‐ and between‐race survival differences.

**Methods:**

Black, White, and Latino individuals diagnosed with nonmetastatic, locally advanced MIBC from 2004 to 2013 within the National Cancer Database were included. Guideline‐based treatment was defined as the receipt including one or more of the following treatment modalities: radical cystectomy (RC), neoadjuvant chemotherapy with RC, RC with adjuvant chemotherapy, and/or chemoradiation based on American Urological Association guidelines. Cox proportional hazards model of mortality estimated effects of GBT status, race/ethnicity, and the GBT‐by‐race/ethnicity interaction, adjusting for covariates.

**Results:**

Of the 54 910 MIBC individuals with 125 821 person‐years of posttreatment observation (max = 11 years), 6.9% were Black, and 3.0% were Latino. Overall, 51.4%, 45.3%, and 48.5% of White, Black, and Latino individuals received GBT. Latino individuals had lower hazard of death compared to Black (HR 0.81, 95% CI 0.75‐0.87) and White individuals (HR 0.92, 95% 0.86‐0.98). With GBT, Latino and White individuals had similar outcomes (HR = 1.00, 95% 0.91‐1.10) and both fared better than Black individuals (HR = 0.88, 95% 0.79‐0.99 and HR = 0.88, 95% 0.83‐0.94, respectively). Without GBT, Latino individuals fared better than White (HR = 0.85, 95% 0.77‐0.93) and Black individuals (HR = 0.74, 95% 0.67‐0.82) while White individuals fared better than Black individuals (HR = 0.87, 95% 0.83‐0.92). Black individuals with GBT fared worse than Latinos without GBT (HR = 1.02, 95% 0.92‐1.14), although not statistically significant.

**Conclusion:**

Low GBT levels demonstrated an “under‐allocation” of GBT to those who needed it most—Black individuals. Interventions to improve GBT allocation may mitigate race‐based survival differences observed in MIBC.

## Graphical abstract

Receipt of guideline‐based treatment nearly eliminated survival disparities between Latino and white individuals with bladder cancer but the benefit was not the same for black individuals, highlighting several potential targets for intervention.

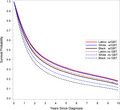

## 1 INTRODUCTION

Racial disparities in bladder cancer in the United States represent the end‐result of a series of “leaks in the pipeline” from diagnosis to treatment. In 2020 alone, an estimated 62 100 incident cases and 13 050 cancer‐specific deaths will be attributed to bladder cancer, with Black individuals presenting with higher stage disease and worse 5‐year survival rates despite lower overall incident rates compared to White individuals.^1^, ^2^ Recent studies focused on race‐based differences in socioeconomic status (SES), quality of care, and treatment for individuals with bladder cancer have highlighted the considerable impact of nonclinical factors on access to quality care and receipt of standard, evidence‐based treatment.^3^, ^4^ Although race and type of treatment have been shown to independently influence outcomes, *how* these two factors may interact to exacerbate racial disparities in bladder cancer is not well understood.

Prior studies have explored racial disparities with respect to survival, often limited to Black‐White comparisons, and generalized the effect to entire racial groups using an unitary approach which focuses on differences by one category alone (ie, race), or a multiplicative approach which layers additional factors, such as SES, on the primary category.^5^ Recent studies have shown Black individuals with muscle‐invasive bladder cancer (MIBC) have 21% lower odds of guideline‐based treatment (GBT) and, in turn, GBT explained 35% of observed Black‐White differences in survival, with an insignificant contribution from inherent tumor characteristics.^3^, ^4^ However, the effects of race and receipt of GBT may interact, resulting in nonadditive (multiplicative) effects. Extending this concept of intersectionality to describe the nonadditivity of microlevel factors such as race and SES with macrolevel factors such as health care implementation or quality of care delivery, is rarely explored.^6^, ^7^ Therefore, it is important to assess whether receipt of GBT modifies racial/ethnic disparities.

Using data from the National Cancer Database (NCDB), a nationwide, facility‐based, oncology outcomes database, we examined how the interaction between race and receipt of GBT impacted survival for Black, White, and Latino individuals with MIBC. By characterizing the intersection of race/ethnicity and treatment delivery, we hope to inform more focused interventions on modifiable factors such as treatment disparities to mitigate race‐based survival differences for individuals with MIBC.

## 2 MATERIALS AND METHODS

Within NCDB, we identified Black, White, and Latino individuals aged 40 years or older who were diagnosed with nonmetastatic, locally advanced MIBC between January 1, 2004 and December 31, 2013. The National Cancer Database, established in 1989, is a joint project of the American Cancer Society and the Commission on Cancer of the American College of Surgeons. The American College of Surgeons has executed a Business Associate Agreement that includes a data use agreement with each of its Commission on Cancer accredited hospitals. The data contained are both publicly available and de‐identified.^8^ Those with nonmuscle invasive bladder cancer, pure CIS, clinical stage Ta, or had clinical evidence of distant metastasis outside of pelvic lymph nodes were excluded. Those treated with palliative treatment were also excluded. The project received exempt status from the University of California, San Francisco institutional review board.

### 2.1 Measures

The primary outcome of interest was overall survival after primary treatment for MIBC. Vital status was reported as the status of the patient (living or deceased) at the time of last observation. Years from treatment initiation to death or last contact (censoring) was used as the time scale variable for survival analyses.

Clinical and demographic variables were included in the multivariable Cox proportional hazards regression model. Age was recorded at the last birthday prior to diagnosis and categorized by decade of life. Sex was recorded at the time of diagnosis as indicated by medical records. Race was defined as Black, White, or Latino based upon self‐report during initial recruitment for NCDB. Small sample sizes limited the ability to disaggregate the Hispanic group, which has been shown to have marked heterogeneity in cancer mortality.^9^ Charlson comorbidity index (CCI) was used as a measure of overall health based on scores of zero, one, or two or greater. Insurance was reported as the primary insurance provider at the time of initial diagnosis and/or treatment. Treatment facilities were classified as one of the following as assigned by the Commission on Cancer Accreditation program to provide a general structural characteristic of each reporting facility: Community Cancer Program (reports 100‐500 new cancer diagnoses annually, may refer for portion of diagnosis or treatment), Academic/Research Program (participates in postgraduate medical education in at least four program areas), Integrated Network (multiple facilities with integrated and comprehensive cancer care) or Comprehensive Community Cancer Program (more than 500 new diagnoses annually with full range of services on‐site or via referral). Clinical TNM staging was defined by the American Joint Committee on Cancer using the Staging Manual edition in use at the time of diagnosis. The reported histologic type was attributed to the most invasive surgical procedure the patient underwent during the study interval. Histology was classified as a urothelial carcinoma or variant. Non‐GBT was defined as receipt of transurethral resection alone or with radiation therapy, primary chemotherapy, cystectomy with radiation therapy, or no treatment. GBT was defined as receipt including one or more of the following treatment modalities: radical cystectomy (RC), neoadjuvant chemotherapy with RC, RC with adjuvant chemotherapy, and/or chemoradiation.

Additional demographic variables were evaluated to further characterize the cohort but were not included in the Cox models. A proxy for patient education level was defined as the percentage of people within a patient's ZIP code who were without a high school diploma using the following categories: less than 7 percent, 7%‐12.9%, 13%‐20.9%, and greater than 21%. A proxy categorical measure of household income corresponded to the percentage of households within a patient's ZIP code in each of the following ranges: less than $38 000, $38 000‐47 999, $48 000‐62 999 or greater than $63 000. Counties were classified into one of three categories based on population size, degree of urbanization, and adjacency to metropolitan areas: Metropolitan, Urban, or Rural.

### 2.2 Summary statistics and Univariate analyses

Descriptive statistics of the study cohort were generated to report demographic, clinical, and pathologic characteristics of the cohort. Means and standard deviations (SD) are reported for continuous variables. Frequencies are reported for categorical variables. Summary statistics were then stratified for comparisons of clinical and pathologic data by GBT status. ANOVA was used to compare means of continuous variables. Chi square test was used to compare frequencies of categorical variables. All tests were adjusted for clustering of individuals within the facility where they received treatment.

### 2.3 Regression models

The primary analysis was a Cox proportional hazards model of time from treatment initiation to death. Survivors' times were censored during the last study observation. Explanatory variables included GBT (yes vs no), patient race/ethnicity, the GBT‐by‐race/ethnicity interaction, sex, age at diagnosis, clinical T stage, clinical N stage, histology type (urothelial carcinoma or variant), insurance status, and treatment facility type. Because of the interaction term, the reported main effect of GBT was averaged across racial/ethnic categories; the reported main effect of race/ethnicity was averaged across GBT status; and simple effects of GBT status within each racial/ethnic category and simple effects of race/ethnicity by GBT status are reported. Clustering of individuals within treatment facilities was accommodated via a random effect. Hazard ratios (HR) are presented with 95% confidence intervals (CI) and *p*‐values. The time scale value was missing for 14.4% of included individuals. All other modeled variables had complete data. Missing values were accommodated via analysis of, and summarization across, 10 multiple imputed datasets. Survival curves stratified by race and GBT status were adjusted for modeled covariates. Statistical analyses were performed using STATA 14.2 (StataCorp, College Station, TX) and SAS/STAT 14.3.

## 3 RESULTS

The study cohort was comprised of 54 910 individuals with clinically staged nonmetastatic MIBC, treated at 1278 unique facilities. Mean observation time from treatment to last contact or death was 2.3 years (range, 0‐11.07) with a total of 125 821.4 person‐years of follow‐up. A total of 38 315 individuals (69.8%) died and 16 595 (30.2%) were censored during the observation period.

Mean age at diagnosis was 72.4 (SD 11.4) with male predominance (71.9%). Of the individuals identified, 6.95% were Black, and 3% Latino. Most were otherwise healthy (CCI of zero, 68.8%; CCI of 1, 22.7%; CCI of 2 or more, 8.5%) with nearly even distributions of household income and education level. Nearly all (70.7%) had government‐funded (Medicare or Medicaid) or private insurance (24.6%) as their primary insurance provider. Most had cT2 stage disease (76.6%), cN0/x (92.9%), and urothelial carcinoma (88.7%). Individuals were most commonly seen at comprehensive cancer centers (45.9%) or academic facilities (35.4%). GBT was more frequently reported at academic facilities (43.5%) followed by comprehensive community centers (40.6%). Overall, half of individuals received GBT (50.9%).

Table 1 shows demographic and clinical characteristics stratified by GBT status. Those who received GBT were younger (<80 years, 82.6% vs 61.1%, *P* < .001), White (91% vs 89.1%, *P* < .001), had urothelial carcinoma (89.6% vs 87.7%, *P* < .001), and had private insurance (28.4% vs 20.6%, *P* < .001) compared to those who received non‐GBT. Individuals who received GBT were most commonly treated at academic facilities (43.5%) while those who received non‐GBT were most commonly treated at comprehensive cancer centers (51.3%, *P* < .001).

**TABLE 1:** Clinical and demographic characteristics by guideline‐based treatment (GBT) status, adjusted for clustering by treatment facility

Characteristic		Overall n (%)	Non‐GBT n (%)	GBT n (%)	*p*
Total		54,910 (100)	26,951 (49.08)	27,959 (50.92)	—
Mean age at diagnosis, years (SD)		72.35 (11.39)	74.94 (11.54)	69.85 (10.65)	
Age (years)	<50	2,154 (3.92)	833 (3.09)	1,321 (4.72)	<.001
	51‐59	7,254 (13.21)	2,820 (10.46)	4,434 (15.86)	
	60‐69	13,021 (23.71)	5,094 (18.90)	7,927 (28.35)	
	70‐79	17,118 (31.17)	7,720 (28.64)	9,398 (33.61)	
	>80	15,363 (27.98)	10,484 (38.90)	4,879 (17.45)	
Sex	Female	15,430 (28.10)	8,295 (30.78)	7,135 (25.52)	<.001
Race	White	49,431 (90.02)	24,002 (89.06)	25,429 (90.95)	<.001
	Black	3,817 (6.95)	2,089 (7.75)	1,728 (6.18)	
	Latino	1,662 (3.03)	850 (3.19)	802 (2.87)	
Charlson Comorbidity Index	None	37,755 (68.76)	18,296 (67.89)	19,459 (69.60)	<.001
	1	12,470 (22.71)	6,079 (22.56)	6,391 (22.86)	
	2+	4,685 (8.53)	2,576 (9.56)	2,109 (7.54)	
Clinical T stage	T2	42,038 (76.56)	20,681 (76.74)	21,357 (76.39)	.34
	T3/T4	12,872 (23.44)	6,270 (23.26)	6,602 (23.61)	
Clinical N stage	No	44,819 (81.62)	21,896 (81.24)	22,923 (81.99)	<.001
	Nx	6,177 (11.25)	3,527 (13.09)	2,650 (9.48)	
	N1	1,844 (3.36)	699 (2.59)	1,145 (4.10)	
	N2	1,766 (3.22)	699 (2.59)	1,067 (3.82)	
	N3	304 (0.55)	130 (0.48)	174 (0.62)	
Histology	Urothelial	48,684 (88.66)	23,644 (87.73)	25,040 (89.56)	<.001
	Variant	6,226 (11.34)	3,307 (12.27)	2,919 (10.44)	
Insurance	Uninsured	1,331 (2.42)	562 (2.09)	769 (2.75)	<.001
	Private	13,493 (24.57)	5,564 (20.64)	7,929 (28.36)	
	Medicaid/Medicare	38,797 (70.66)	20,129 (74.69)	18,668 (66.77)	
	Other/Unknown	1,289 (2.35)	696 (2.58)	593 (2.12)	
Household income	< $38,000	10,180 (18.54)	5,274 (19.57)	4,906 (17.55)	<.001
	$38,000 ‐ $47,999	13,576 (24.72)	6,567 (24.37)	7,009 (25.07)	
	$48,000 ‐ $62,999	14,718 (26.80)	7,112 (26.39)	7,606 (27.20)	
	$63,000+	16,436 (29.93)	7,998 (29.68)	8,438 (30.18)	
% with < HS education	>=21%	9,542 (17.38)	4,925 (18.27)	4,617 (16.51)	<.001
	13% ‐ 20.9%	14,242 (25.94)	6,957 (25.81)	7,285 (26.06)	
	7% ‐ 12.9%	18,583 (33.84)	9,074 (33.67)	9,509 (34.01)	
	<7	12,543 (22.84)	5,995 (22.24)	6,548 (23.42)	
Facility type	Community Cancer Program	6,702 (12.21)	4,062 (15.07)	2,640 (9.44)	<.001
	Comprehensive Community Cancer Program	25,176 (45.85)	13,827 (51.30)	11,349 (40.59)	
	Academic/Research Program	19,449 (35.42)	7,298 (27.08)	12,151 (43.46)	
	Integrated Network Cancer Program	3,529 (6.43)	1,751 (6.50)	1,778 (6.36)	
	Other	54 (0.10)	13 (0.05)	41 (0.15)	
Years of follow‐up, mean (SD)		2.39 (2.29)	2.10 (2.31)	2.68 (2.24)	

**FIGURE 1:**
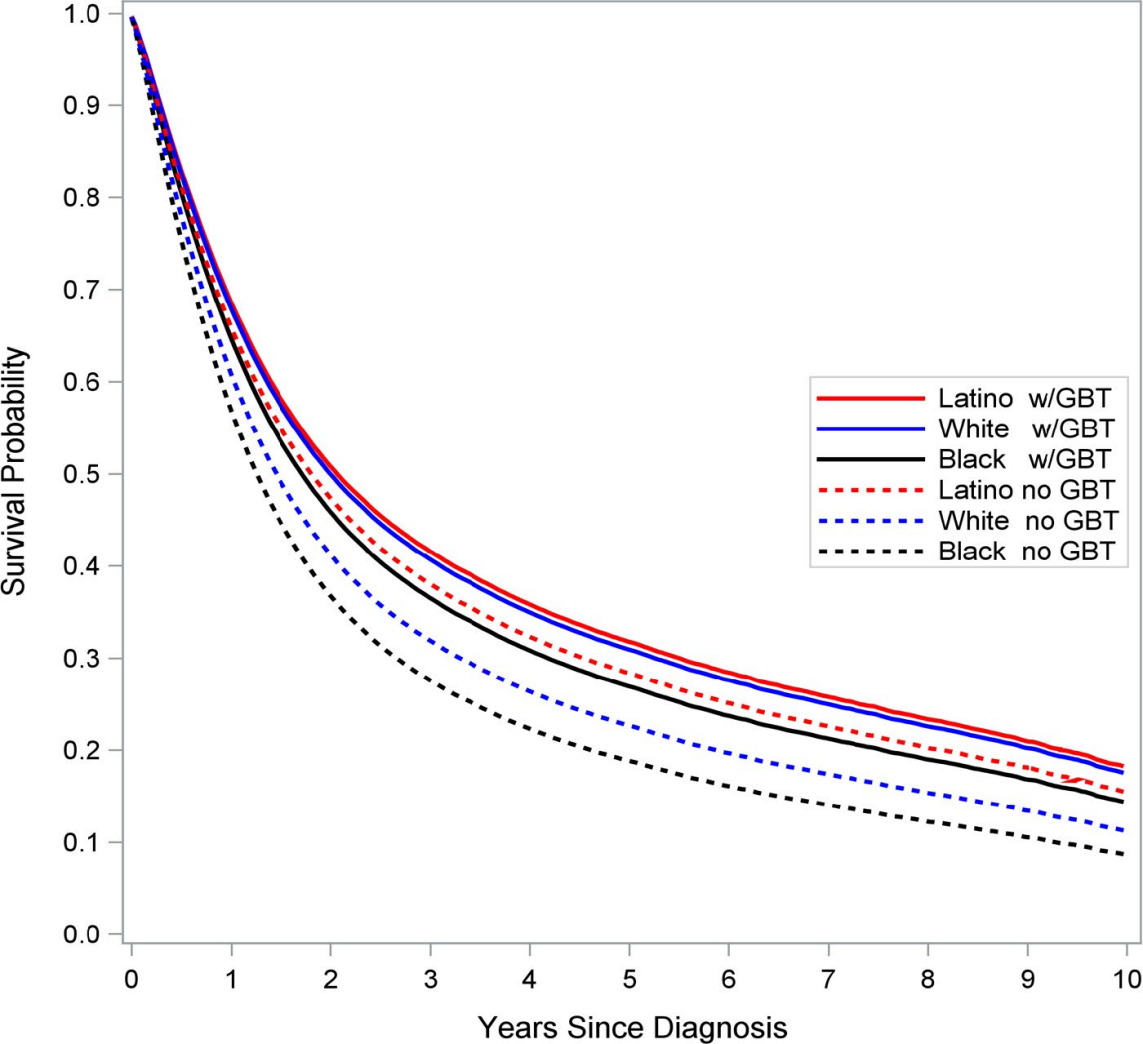
Adjusted overall survival curve estimates stratified by guideline‐based treatment (GBT) status and Race/Ethnicity

Averaging across racial groups, compared to those who did not receive GBT, individuals who received GBT had lower mortality hazard (HR = 0.81, 0.77‐0.85; Table 2). Averaging across GTB status, Black individuals had higher mortality hazard compared to their White and Latino counterparts (HR = 1.14, 1.09‐1.19 and HR = 1.24, 1.15‐1.33, respectively), while White individuals had higher mortality hazard compared to Latino individuals (HR = 1.09, 1.02‐1.16). Figure 1 shows the multivariable Cox model‐adjusted survival estimates up to 10 years after treatment, stratified by race/ethnicity and GBT status. A similar pattern of relative hazards was obtained when restricting to individuals who did not receive GBT: Black versus White and Latino individuals (HR = 1.14, 1.08‐1.21 and HR = 1.35, 1.22‐1.50, respectively); and White versus Latino individuals (HR = 1.18, 1.08‐1.29). However, because of a significant GBT‐by‐race/ethnicity interaction effect (*P* = .0456) a different pattern of relative hazards resulted among those who received GBT: Black individuals continued to have higher mortality risk compared to White and Latino individuals (HR = 1.13, 1.06‐1.20 and HR = 1.13, 1.02‐1.26, respectively), yet the relative hazard across White and Latino individuals was roughly equivalent (HR = 1.00, 0.91‐1.10). One additional comparison helps to highlight a key finding: Black individuals who received GBT had roughly equivalent mortality risk as Latino individuals who did *not* receive GBT (HR = 1.02, 0.92‐1.14).

**TABLE 2:** Predictors of overall survival using Cox proportional hazards

Variable		HR	95% LL	95% UL	*p*
Age groups	<50	Ref	—	—	—
	50‐59	1.09	1.01	1.17	.03
	60‐69	1.20	1.12	1.28	<.001
	70‐79	1.50	1.40	1.61	<.001
	>80	2.32	2.16	2.49	<.001
Female (vs male)		1.06	1.03	1.08	<.001
Charlson comorbidity index	0	Ref	—	—	—
	1	1.22	1.19	1.26	<.001
	2+	1.59	1.51	1.62	<.001
Insurance	None	Ref	—	—	—
	Private	0.76	0.71	0.92	<.001
	Government funded	0.92	0.84	0.99	.02
	Other/unknown	0.89	0.80	0.98	.02
Variant histology (vs urothelial carcinoma)		1.27	1.23	1.31	<.001
cT stage III‐IV (vs II)		1.34	1.31	1.38	<.001
cN stage	N0	Ref	—	—	—
	Nx	1.13	1.10	1.17	<.001
	N1	1.49	1.41	1.58	<.001
	N2	1.71	1.62	1.81	<.001
	N3	1.92	1.68	2.19	<.001
Facility type	Community hospital	Ref	—	—	—
	Comprehensive cancer center	0.98	0.95	1.02	.43
	Academic/research center	0.94	0.90	0.98	.01
	Integrated Network	1.02	0.95	1.10	.51
	Other	1.20	0.77	1.85	.42
Race (main effect)^a^	Black vs White	1.14	1.09	1.29	<.001
	Black vs Latino	1.24	1.15	1.33	<.001
	White vs Latino	1.09	1.02	1.16	.01
GBT (vs non‐GBT; main effect)^b^		0.81	0.77	0.85	<.001
Race‐by‐GBT interaction effect *Simple effects of Race within GBT strata* ^c^					.046
Black vs White (with GBT)		1.13	1.06	1.20	<.001
Black vs Latino (with GBT)		1.13	1.02	1.26	.03
White vs Latino (with GBT)		1.00	0.91	1.10	.98
Black vs White (with non‐GBT)		1.14	1.08	1.21	<.001
Black vs Latino (with non‐GBT)		1.35	1.22	1.50	<.001
White vs Latino (with non‐GBT)		1.18	1.08	1.29	<.001

^a^ Because the model included a significant race‐by‐GBT status interaction effect, these HR estimates reflect race comparisons that are averaged over GBT status.

^b^ this HR estimate reflects the GBT effect averaged across racial groups.

^c^ The simple effects of race within GBT strata characterize the nature of the Race‐by‐GBT interaction effect.

## 4 DISCUSSION

Generally, Black individuals had poorer MIBC survival than their White and Latino counterparts and those disparities persisted when GBT was provided. In contrast, the general survival advantage observed for Latino patients compared to their White counterparts was eliminated when GBT was provided. Our findings emphasize the significant reduction in mortality risk conferred with receipt of GBT, yet utilization is low in vulnerable populations such as Black individuals, highlighting an actionable target to improve survival for individuals from diverse backgrounds diagnosed with MIBC.

GBT for MIBC is not widespread, as only half (50.92%) of the cohort received guideline‐concordant care. Although concerning, our finding is not unique. Low rates of GBT have been observed in other populations with bladder cancer and have been associated with several factors including sex, race, geographic/regional resources, and health care costs. A recent study from Sweden reported that, despite a slowly increasing incidence of MIBC, approximately half (57%) the individuals with MIBC received treatment with curative intent.^10^ Specifically for race, we previously reported that Black individuals had 21% lower odds of receiving GBT compared to White counterparts with the same stage disease treated in the same facility.^4^ Additionally, Black individuals may receive different treatments and were more likely to receive radiotherapy rather than radical cystectomy alone compared to other groups.^11^ While the study found that distance to treatment facility did not affect compliance with most care measures, others have shown that an individual living farther from treatment facilities may be exposed to greater limitations on health care access and increased health care spending.^12^ Although these studies focus on less advanced disease, the rates of GBT in these studies are consistent with our findings and provide further evidence of how nonclinical factors impact the quality of care received and subsequent clinical outcomes. In this study, Black individuals experienced increased mortality risk compared to White and Latino counterparts, even when receiving GBT. Therefore, provision of GBT alone may be insufficient to address racial/ethnic disparities in Black versus White/Latino survival. These findings of the survival benefits of GBT and the increased mortality risk experienced by Black individuals with MIBC highlight the need for more focused and tailored interventions specific to Black individuals in order to reduce racial disparities in individuals with MIBC.

Overall, the GBT effect improved survival both in within‐ and between‐racial groups, yet this survival benefit was variable. GBT reduced the risk of death by 28%‐29% for White and Black individuals and 15% for Latino individuals, which may be due to Latino individuals having relatively better outcomes even without GBT and thus have less room for improvement. These findings are important because it demonstrates how GBT can mitigate the race‐based survival disparities previously reported and explores outcomes in a population not typically included in such studies. As most studies are restricted to Black and White individuals, less is known about how race‐based treatment disparities impact survival for Latino individuals with bladder cancer. Yee et al, examined race‐based differences in disease‐specific survival for individuals diagnosed with urothelial carcinoma of the bladder and demonstrated that 5‐year disease‐specific survival was similar between White and Latino individuals and both were higher than that of Black individuals (Black 70.2%; White 82.8%; Latino 80.7%).^11^ Treatment did differ by race, as Black individuals were less likely to receive surgery and more likely to receive radiotherapy, but the influence of these treatment differences on survival was not explored further. Our findings paint a similar picture and show not only race‐based differences in overall survival, but also survival differences within GBT strata. Cole et al, also explored the influence of race in the outcome of cT2‐4NoMo individuals undergoing radical cystectomy in NCDB and used an inverse‐probability of treatment weighting approach to show that Black‐White differences existed in both the quality of surgical care received, location of treatment (hospital volume variation), and overall survival.^3^ Although similar in focus, our study explores this issue further by assessing the impact of treatment disparities both within‐ and between‐race groups while expanding the cohort to include Latino individuals.

Our study has several strengths and limitations which should be addressed. First, the NCDB dataset allowed for a large study cohort but was not restricted only to individuals with locally advanced, node‐negative MIBC as prior studies have done in the past. Although our study focused on overall survival compared to disease‐specific survival, a significant proportion of deaths in the short‐term have been shown as likely attributable to cancer‐specific mortality.^13^ By focusing on more advanced disease and the appropriate respective treatments, we have improved generalizability and clinical relevance to a cohort with greater potential benefit if quality care is received. The dataset used for this study does not provide information on how and by whom treatment decisions were made but by clustering at the facility level we attempted to account for nuanced facility‐specific differences in practice which would otherwise be unmeasured in the analysis. Although more than 1600 Latino patients are included in this analysis, the proportion of the total cohort remains low, consistent with other databases currently used. To our knowledge, this study is one of the first to utilize multiple imputation to address missing time scale values and ensuing potential bias. Lastly, our study does include Latino individuals with bladder cancer but was unable to disaggregate this specific group due to the lack of granular ethnicity data. Disaggregation in this heterogenous population has been previously shown to elucidate significant variations in cancer mortality which could be informative for future cancer control and prevention efforts.^9^ Despite this, selective comparisons in our analysis do provide a detailed characterization of the GBT effect in Latino individuals, who were not previously included in similar research studies, while expanding the findings of outcomes to more than Black‐White comparisons. As a result of using this intersectional approach, we were able to show that simply estimating main effects of race/ethnicity and treatment does not accurately reflect their combined impact on MIBC.

## 5 CONCLUSION

Our study illustrates that GBT modifies the effect of race on survival for individual with MIBC. The GBT effect was not uniform across groups with a 28%‐29% reduction in mortality risk experienced by White and Black individuals and 15% reduction for Latino counterparts. The receipt of GBT represents an actionable target for interventions to improve survival for individuals from diverse backgrounds diagnosed with MIBC and remains a factor directly impacted by urologic providers which may mitigate the race‐based survival differences observed in individuals with MIBC.

## CONFLICT OF INTEREST

The content is solely the responsibility of the authors and does not necessarily represent the official views of the National Institutes of Health. The authors declare no potential conflicts of interest.

## AUTHOR CONTRIBUTIONS

Samuel Washington: Conceptualization, data curation, funding acquisition, writing—original draft, and writing—review and editing. Steven Gregorich: Conceptualization, data curation, formal analysis, software, methodology, visualization, writing—review and editing. Maxwell Meng: Conceptualization, writing—review and editing, supervision. Anne Suskind: Conceptualization, funding acquisition, writing—review and editing, supervision. Sima Porten: Conceptualization, funding acquisition, writing—review and editing, supervision.

## Data Availability

The data that support the findings of this study are openly available in the National Cancer Database at https://www.facs.org/quality‐programs/cancer/ncdb.
